# Secretion of Extracellular Microvesicles Induced by a Fraction of *Escherichia coli*: Possible Role in Ovarian Cancer with Bacterial Coinfections

**DOI:** 10.3390/ijms262110653

**Published:** 2025-11-01

**Authors:** Francisco Sierra-López, Juan Carlos Fernández-Hernández, Lidia Baylón-Pacheco, Verónica Ivonne Hernández-Ramírez, Juan Carlos Bravata-Alcántara, Vanessa Iglesias-Vázquez, Susana Bernardo-Hernández, Daniel Medrano-Espinosa, Gustavo Acosta-Altamirano, Patricia Talamás-Rohana, José Luis Rosales-Encina, Mónica Sierra-Martínez

**Affiliations:** 1 Department of Infectomics and Molecular Pathogenesis, Center for Research and Advanced Studies, Av. Instituto Politécnico Nacional 2508, Zacatenco, Mexico City 07360, Mexico; luck19861990@gmail.com (F.S.-L.); ptr@cinvestav.mx (P.T.-R.); 2 Health Research Unit, Hospital Regional de Alta Especialidad de Ixtapaluca, Servicios de Salud del Instituto Mexicano del Seguro Social para el Bienestar (IMSS-BIENESTAR) México, Carr Mex-Puebla Km 34.5 col., Zoquiapan, Mexico City 56530, Mexico; juan.fernandez@salud.gob.mx (J.C.F.-H.); vanessa.iglesias2711@gmail.com (V.I.-V.);; 3 Hospital General de México, Eje 2A Sur (Dr. Balmis) No. 148, Cuauhtémoc, Doctores, CDMX, Mexico City 06726, Mexico; 4 Department of Oncology, Hospital Regional de Alta Especialidad de Ixtapaluca, Servicios de Salud del Instituto Mexicano del Seguro Social para el Bienestar (IMSS-BIENESTAR) México, Carr Mex-Puebla Km 34.5 col., Zoquiapan, Mexico City 56530, Mexico

**Keywords:** EVs, ovarian cancer, PEVs, SKOV-3 line

## Abstract

Ovarian cancer (OC) is usually diagnosed at an advanced stage, contributing to its high mortality rate. The presence of concurrent bacterial infections in these patients is a common clinical observation, and the mechanisms by which this coinfection influences tumor progression are still not fully understood. This study investigates the role of polydisperse extracellular vesicles (PEVs) secreted by OC cells in response to bacterial components, aiming to elucidate a potential communication pathway between OC and the bacterial microenvironment. We stimulated a human OC cell line in vitro with a fraction of *E. coli*. Our results show that this bacterial stimulation significantly increases the secretion of PEVs by cancer cells. A subsequent proteomic analysis of these PEVs revealed an enrichment of proteins, including filamin A, filamin B, alpha-enolase, and heat shock cognate 71 kDa protein. In addition, the PEVs displayed protease activity (on fibronectin and gelatin) and phosphatase activity against para-nitrophenyl phosphate, indicating their capacity to alter cellular signaling. This represents a novel mechanism through which bacterial coinfection may influence the biological behavior of OC if bacteria interact with tumor cells, potentially contributing to their aggressiveness and the challenges associated with their treatment. Our work highlights the importance of studying the interplay between the tumor and its associated microbiota to better understand ovarian cancer progression and identify new therapeutic targets.

## 1. Introduction

Ovarian cancer (OC) is one of the most lethal gynecological cancers, often diagnosed at advanced stages due to its asymptomatic nature in the early stages [1]. Despite therapeutic advances in treatment, the five-year survival rate remains low, highlighting the urgent need for continued research into the molecular mechanisms and interactions that drive its progression [2].

The interplay between microorganisms and carcinogenesis has become a focus of current research, as creating bacteria can amplify carcinogenic effects and create a microenvironment that favors cell transformation [3]. These microorganisms may accelerate cancer progression by promoting angiogenesis and metastasis. For instance, in colorectal cancer, the bacterium *Fusobacterium nucleatum* has been associated with more aggressive disease and a poorer response to chemotherapy [4,5]. Additionally, bacteria can modulate the tumor microenvironment (TME) by suppressing the antitumor immune response, thereby facilitating tumor escape [6].

Bacteria such as *Mycoplasma hominis*, *Chlamydia trachomatis*, and *Escherichia coli* (*E. coli*) have been reported in OC, implying their possible implication in chronic inflammation and co-carcinogenesis [7,8]. Among biomarkers of risk for ovarian cancer [9], bacteria have been suggested that could be influencing tumor development, such as *Escherichia coli* [3,10,11,12]. Among the bacterial species in the microbiome of vaginal and cervical samples that have been reported as prominent or enriched in patients with OC are those belonging to the genera *Dialister* (Gram-negative), *Prevotella* (Gram-negative), *Corynebacterium* (Gram-positive), and *Peptoniphilus* (Gram-positive), and the enriched specific taxa in low- and high-grade patients compared to the benign cohort include *Streptococcus infantis*, *Fusobacterium nucleatum*, *Varibaculum cambiense*, *Escherichia coli*, *Faecalibacterium prausnitzii*, and *Bacteroides fragilis* [13]. Gram-negative bacteria, including *E. coli*, are among the most common pathogens found in cancer patients [14,15,16], including those with OC [17]. Notably, *E. coli* has been shown to modulate tumor progression across various tissues [3,10,11,12,17]. Among the toxins produced by some strains of *E. coli*, colibactin has been linked to colorectal cancer and DNA damage [10,18]. Recently, OmpF and OmpC from *E. coli* have been associated with the ability to form toxic fibrillar aggregates with amyloid properties that could contribute to cancer pathogenesis [19].

Structural components of Gram-negative bacteria, such as lipopolysaccharide (LPS), contribute to chronic inflammation and carcinogenesis [20], as well as other outer membrane proteins, such as OipA from *Helicobacter pylori* (*H. pylori*) [21]. CagA is another toxin produced by *H. pylori* in the gastric epithelial cells, which has a key role in gastric cancer, causing dysregulation of signaling pathways, cell proliferation, adhesion, and migration [6].

It is currently known that pathogens can interact with target cells without the need for direct contact, through the secretion of biomolecules and extracellular vesicles (EVs) that can travel through biological fluids, making it possible for them to exert an effect on distant organs and cells [22]. Gram-negative bacteria have the ability to use their own EVs called ‘outer membrane vesicles’ to carry components of the bacterial outer membrane such as LPS and OMPs, delivering the cargo to tumor cells and immune cells (carrying out interactions between receptors and ligands, PAMPs-PRRs) in the microenvironment, affecting their proliferation, migration and metastatic capacity [23].

Intercellular communication mediated by EVs has emerged as a key player in cancer pathogenesis, metastasis, and chemotherapy resistance [24,25]. EVs are heterogeneous nanostructures that are commonly spherical in shape, with at least one lipid bilayer delimiting them. They are subdivided based on size and biogenesis into (i) exosomes, (ii) microvesicles, and (iii) apoptotic bodies. Polydisperse extracellular vesicles (PEVs) are so-called based on their sizes [26,27].

Biogenesis of EVs is distinguished by their subcellular origin: exosomes (40–150 nm) originate in the endosomal system through the release of intraluminal vesicles from multivesicular bodies; microvesicles or ectosomes (100–1000 nm) bud directly from the plasma membrane; and apoptotic bodies (800–5000 nm) originate from cells undergoing apoptosis [24,28]. There are also subtypes, such as large oncosomes (0.5–10 µm) [29,30], which are a subtype of large microvesicles released by tumor cells through plasma membrane shedding and are key in cancer progression [31,32].

EVs are released by both healthy cells and tumor cells [24], carrying a wide variety of molecules, such as proteins, lipids, and nucleic acids, which facilitate the crosstalk between parental and recipient cells [31,33,34]. The EVs secreted by tumors act as cell–cell mediators [24]. Proteomic analysis, such as that of pancreatic EV samples, has identified that cancer EVs contain a distinctive protein profile, where tumor-promoting candidates such as LAMA5 (laminin subunit alpha 5), SDCBP (Syndecan Binding Protein) and TENA (Tenascin-A) were found to be overexpressed [25]. Among other molecules that have been identified as cargo in EVs involved with cancer are integrin B1 that mediates EV uptake and RNA delivery [35], fibronectin and the induction of its assembly [35,36], osteopontin involved in the signaling process [37], cytokines that modulate metabolism and tumor microenvironment [33], and Hsp70, which is commonly found in small EVs [34].

The tumor microenvironment commonly contains a high concentration of PEVs, and tumor cells have been observed to be able to secrete a higher amount of EVs compared to healthy cells; for example, more PEVs are secreted from pancreatic cancer organoids versus healthy pancreas organoids [31]. Furthermore, it has been observed that more aggressive tumor cells secrete a significantly greater quantity and variety of PEVs than less aggressive cells [24,29,30,38]. This increased secretion of PEVs is therefore considered to be a key mechanism by which cancer cells transmit their malignant characteristics and remodel the environment to their advantage [32,33].

We have previously reported that a fraction of *E. coli* bacteria is able to induce macrophages to release abundant PEVs, using the LMW-PTP (ACP1) protein as a marker for them and monitoring them using confocal microscopy when large PEVs are present [26]. Therefore, we have used the induction strategy, and this study focuses on investigating whether a fraction of *E. coli* is capable of inducing PEV secretion in SKOV-3 ovarian cancer cells, which could be reflected in the abundance of PEVs, and could even lead to the secretion of those PEVs that allow their monitoring by confocal microscopy. We have also tested the biological activity of PEVs in terms of key functions such as proteolytic activity and phosphatase enzyme activity. Our findings could offer a new perspective on the pathogenesis of this disease and the role of bacterial infections as stimulants to cancer cells with phenotypes related to greater aggressiveness, and also provide a possible alternative to obtain in vitro the varieties of PEVs that these cancer cells could be producing in vivo.

## 2. Results

### 2.1. Electrophoretic Profile of SKOV-3 Extracellular Vesicles Induced by an E. coli Fraction

**Figure 1:**
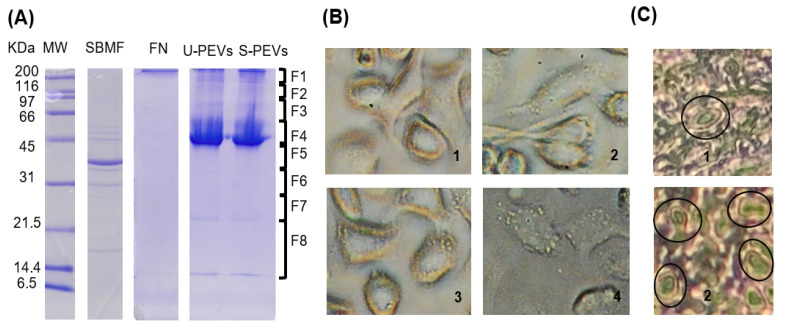
A fraction of Gram-negative *E. coli* bacteria induces increased secretion of PEVs from SKOV-3 ovarian cancer cells. (**A**) The 12% SDS-PAGE results of protein profiles of U-PEVs (20 µg of unstimulated PEVs) and S-PEVs (20 µg of PEVs from stimulated cultures). SBMF (SDS-soluble *E. coli* membrane fraction) was placed as a substrate together with FN (human fibronectin) on the surfaces of cell cultures as a stimulant. MW: molecular weight. (**B**) Appearance of SKOV-3 cells after 2 h of unstimulated (1) or stimulated (2–4) culture. Multiple large EV-like particles are observed on the surface of cells in 2–4. (**C**) Histological sections of healthy ovary (1) and ovarian cancer (2) showing regions with several cells with large EV-like membrane protrusions (ovals), whereas these were rarely found in healthy ovary tissue.

An electrophoretic analysis was performed to characterize the extracts and vesicles used in the study. In the SDS-PAGE gel (Figure 1A), column 2 shows the bacterial extract with the expected profile, which, according to the description of this fraction reported previously, is mainly composed of *E. coli* outer membrane proteins, which is why we named the fraction “SBMF,” and it is soluble in an SDS solution (Supplementary Materials, Table S1). The most intense band of SBMF corresponds to the 39.3 kD OmpF protein. Other proteins were also identified, such as OmpA (37.2 kD), OmpC (53.7 kD), ATP synthase subunits (atpD of 50.3 kD, atpA of 55.2 kD), maltoporin LamB (49.9 kD), several lipoproteins, and other outer membrane proteins (OmpX, Lpp, RcsF) [26]. The presence of these proteins suggests that the fraction corresponds mainly to the bacterial outer membrane, containing lipopolysaccharide (LPS) and bacterial lipids. Column 3 exhibited human fibronectin, identified as a single band above 200 kDa, which was used as an agent to stably distribute and adhere the bacterial fraction to cell culture surfaces. Column 4 showed the profile of polydisperse extracellular vesicles (PEVs) obtained from unstimulated cells, with a main band near 45 kDa, and other bands above 45 kDa and even above 200 kDa, as well as between 14 and 21.5 kDa. Column 5, with vesicles from stimulated cells, revealed a similar profile, but with more intense bands.

Using an inverted light microscope (Figure 1B), the morphology of SKOV-3 cells was observed. Unstimulated cells (Figure 1(B1)) showed no vesicles, as is commonly observable by this technique. In contrast, stimulated cells (Figure 1(B2–4)) displayed a large number of extracellular vesicles anchored to their surface. Cell morphology varied, ranging from elongated cells (Figure 1(B2)), suggesting migration, to more oval and plump cells (Figure 1(B3)).

Histological analysis of ovaries (Figure 1C) revealed the presence of large extracellular vesicle-like protrusions. After searching, cells with these structures were rarely identified in the healthy ovary section (Figure 1(C1)), while in the cancerous ovary section, regions were found where multiple cells displayed these protuberances (Figure 1(C2)).

### 2.2. Localization and Quantification of LMW-PTP

**Figure 2:**
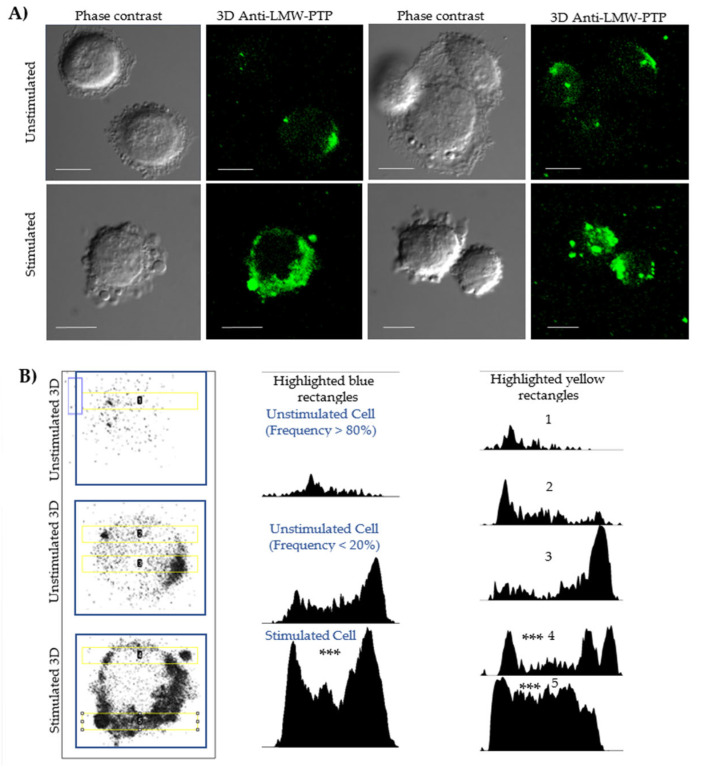
Confocal microscopy and densitometry of the increase in LMW-PTP expression during stimulation of the secretion of PEVs in SKOV-3 ovarian cancer cells. (**A**) SKOV-3 cells were stimulated or not stimulated with SDS-SBMF-FN extract from *E. coli* bacteria, and after 2 h, they were analyzed by confocal microscopy. The observation of multiple large EVs increased in stimulated cells, some of which contained LMW-PTP (green—ITC); LMW-PTP labeling was also polarized in the plasma membranes and cytoplasm. Bar scale 10 µm. (**B**) Images of SKOV-3 cells in the left column in grayscale with 3D recognition of LMW-PTP; central column: densitometry corresponding to the amount of LMW-PTP analyzed at the cell scale (blue box); right column: densitometry of a 20 µm long rectangular region (yellow) that takes as many particles similar to large EVs and a portion with LMW-PTP labeling from the cells. It was analyzed with one-way ANOVA and the post hoc Bonferroni test, representing the difference between stimulated vs. frequent appearance of unstimulated cells (*n* = 4); *** *p* < 0.001 as significant.

Fluorescence microscopy and densitometry analyses evaluated the localization and any increase in protein related to cells and PEVs (Figure 2, Supplementary Materials, Table S2). An antibody against LMW-PTP was used because previous studies have shown that this same bacterial fraction induces the secretion of PEVs enriched with this phosphatase in macrophages. In our model, unstimulated cells showed a distribution of LMW-PTP in the cytoplasm, close to the membrane (Figure 2B, column 1, row 1), with a polarization that is sometimes more visible in cells (Figure 2B, column 1, row 2). Densitometry of unstimulated whole cells (Figure 2B, column 2, row 1) showed that they commonly have a low LMW-PTP intensity.

In stimulated SKOV-3 cells (Figure 2A, row 2 Stimulated), a significant increase in the number of PEVs was observed. Although not all vesicles showed labeling, the intensity of LMW-PTP was significantly increased in specific populations, which was reflected in a higher signal intensity in the densitometry of stimulated cells (Figure 2B, column 2, row 3). Densitometry analyses in a 20 µm long strip (Figure 2B, column 3) validated that the peaks of highest LMW-PTP labeling intensity were coincident with the location of PEVs and the cell periphery and revealed a statistically significantly different pattern comparing the most frequent form of unstimulated cells with stimulated cells secreting PEVs.

### 2.3. Enzymatic Activity of EV

**Figure 3:**
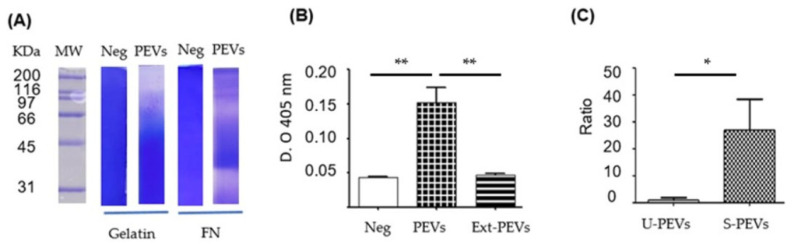
Biological activity in SKOV-3 PEVs induced with FN-SBMF *E. coli*. (**A**) Zymograms of gelatin or FN as substrate, where the total extract of SKOV-3 PEVs induced with SDS-SBMF was run. (Neg) control without load. (**B**) Absolute phosphatase activity at 2 h with 1.6 µM pNPP; PEVs were pre-activated with DTT. (Neg) control without sample, (PEVs) unlysed PEVs, (Ext-PEVs) PEV extract by sonication. (**C**) PEV secretion ratio taken as Ratio 1 to the secretion detected with EVAnalyzer in unstimulated cells. U-PEVs: unstimulated; S-PEVs: stimulated. *n* = 3 (**B**) and *n* = 6 (**C**) were used for one-way Anova with Bonferroni’s post hoc test, * *p* < 0.05, ** *p* < 0.01.

The activity of the vesicles was demonstrated using enzymatic assays (Figure 3). Zymography (Figure 3A) showed proteolytic activity against gelatin (column 3) and fibronectin (column 5). With gelatin as a substrate, intense proteolytic activity was shown at weights above 116 kDa and subtle activity between 50 and 116 kDa. With FN as a substrate, intense proteolytic activity was shown in different regions from around 31 kDa to above 200 kDa, mainly close to 31–35 and 66 kDa. The phosphatase activity assay (Figure 3B) revealed that the PEVs extract (column 3) showed no activity, similar to the negative control without PEVs, but where intact PEVs were placed, there was greater catalytic activity, indicating the possibility of phosphatase degradation in the total extracts of these PEVs.

### 2.4. Proteomic Profile of EVs

Mass spectrometry analysis identified the proteins present in PEVs obtained under induction with the membrane fraction of *E. coli* Gram-negative bacteria. Key proteins such as filamin A-B, alpha-enolase, heat shock cognate 71 kDa protein, tubulin, actin, ezrin, heat shock 70 kDa protein 1A/1B, pyruvate kinase PKM, vimentin, albumin, elonfation factor 1-alpha 1, peptidyl-prolyl cis trans isomerase A, annexin A1–5, heat shock protein HSP 90, phosphoglycerate kinase 1, spliceosome RNA helicase DDX39B, moesin, clathrin, peroxiredoxin 1, glyceraldehyde-3-phosphate dehydrogenase, fibronectin, alpha-2-macroglobulin, protein disulfide-isomerase A3, L-lactate dehydrogenase A chain, and radixin were found, among others with lower ‘Unused’ identification (Supplementary Materials, Table S3). The presence of LMW-PTP phosphatase was confirmed, although at a low concentration that resulted in a low ‘Unused’ value, which may be related to the degradation observed above.

## 3. Discussion

The role of PEVs in intercellular communication and cancer progression is a growing area of research. Traditionally, attention on EVs has focused on the smallest ones originating in multivesicular bodies and released by the endosomal pathway, known as exosomes (30–150 nm) [34,39], followed by EVs released by evagination of the plasma membrane, also known as microvesicles or ectosomes, whose dimensions can vary greatly, from a few tens of nm to several micrometers [28]. In recent years, it has been shown that cancer cells can release both small and large EVs of several micrometers in size, with characteristics specific to cancer cells, which is why they have also been given the names oncosomes and large oncosomes, due to the role of these populations of PEVs in each group within cancer biology [28,30]. In addition, these samples of oncosomes, mainly large oncosomes or PEVs, can provide markers for liquid biopsies [27,29].

As mentioned in the introduction, some microorganisms can trigger cancer or aggravate it if it is already present when they infect people. These include bacteria, among which species have been found that can enhance carcinogenic effects and create a microenvironment more favorable to cell transformation [3], increasing cancer progression and metastasis, as is the case with *Fusobacterium nucleatum* in colorectal cancer [4,5,40], *Helicobacter pylori* in gastric cancer [6], or increasing inflammatory damage as with *Mycoplasma hominis* and *Chlamydia trachomatis* in ovarian cancer [7]. *Escherichia coli* is among the bacterial species in the microbiome that have been suggested to influence cancer development in various tissues [10,12], being among the most prevalent Gram-negative bacteria in cancer patients [14,15,16]. Among the molecules and outer membrane proteins of Gram-negative bacteria that aggravate cancer or favor cancer cells in the microenvironment are lipopolysaccharide (LPS) [20] and outer membrane proteins such as OipA [21], OmpF, OmpC [19].

Our results demonstrate that the fraction we used from the outer membrane of Gram-negative *E. coli* bacteria contains a mixture of molecules that act as a potent inducer of EV secretion in SKOV-3 ovarian cancer cells. We previously described and reported this fraction as a strong inducer of the secretion of a wide variety of polydisperse EVs (PEVs) secreted by macrophages, which contain various porins, among other proteins, and are suggested to contain lipopolysaccharide (LPS) and lipids [26]. It has been documented that LPS is capable of inducing an increase in small EVs (exosomes) from human cells [37]. Likewise, subcellular fractions such as vesicles secreted by bacteria (called ‘outer membrane vesicles’) from *Porphyromonas gingivalis* [41] and *Helicobacter pylori* [42], which contain porins and LPS, are capable of inducing the general secretion of EVs into host cells. Furthermore, in our case, human fibronectin (FN), which was used to stabilize the bacterial stimulus, may have contributed to the response by acting as an adhesion substrate that would facilitate the interaction between SKOV-3 cells and the bacterial fraction of *E. coli*, helping to observe stimulated cells secreting PEVs at low doses. Then, in the membrane fraction that we have used for the stimulation of cells to secrete EVs, there are components that separately and in combination have been reported as inducers of EV secretion, which suggests that we have obtained a stimulant with components that could be acting with synergistic effects, and in turn suggests that these bacterial components could stimulate robust secretion of EVs to various types of tumor cells, a characteristic that is associated with aggressive cancer, which opens the possibility to take it for future studies.

A relevant finding of our study is the presence of LMW-PTP phosphatase in some EV populations, which increase in quantity after cell stimulation. This is particularly relevant because the previous study with the same bacterial fraction showed that it induces the production of LMW-PTP-enriched EVs in macrophages and increases the expression of this protein in both the cytoplasm and plasma membrane [26]. The detection of LMW-PTP in some populations or subpopulations of SKOV-3 EVs suggests that the induction of phosphatase and its packaging into vesicles is a conserved response mechanism in different cell types for some EV populations, not limited to immune system cells. This reasoning is supported by the report of the identification of LMW-PTPs in small EVs from colorectal cancer cells [43].

Analysis of the enzymatic activity of EVs revealed a crucial aspect the loss of phosphatase activity when lysing EVs. This phenomenon, coupled with the low ‘Unused’ LMW-PTP in mass spectrometry, suggests that proteases, also encapsulated in EVs, degrade both LMW-PTPs and other enzymes once the vesicular membrane is broken or when the integrity of the EVs is lost. Therefore, these EVs are not simply a transport vehicle, but a protective compartment that maintains an order that allows enzymes susceptible to degradation to be maintained in the extracellular microenvironment. The self-degradation of proteins in total extracts from some EV populations by their own proteases is an event that has been documented [44].

The detailed proteomic profile of EVs shows the presence of proteins associated with the cytoskeleton and vesicular biogenesis, such as actin, vimentin, annexins, and clathrin, supporting the idea that a portion of the mixture of EV-like particles are formed by the evagination of ectosomes or microvesicles from the plasma membrane. The identification of glycolytic enzymes (such as pyruvate kinase and alpha-enolase) and heat shock proteins (HSP70/90) in EVs suggests a possible state of metabolic stress in SKOV-3 cells, as has been observed in other cancer cells [45]. The presence of fibronectin in EVs could be explained by the internalization of substrate fibronectin by SKOV-3 cells, which loaded the glycoprotein into EVs, or by the fact that once secreted, EVs captured it from the cell culture surface where it was located together with the stimulus. The uptake and incorporation of proteins from the extracellular medium into EVs have been previously documented [35,36]. Additionally, the detection of albumin in EVs can be explained by the fact that the cells were previously cultured in a medium with fetal bovine serum, internalized and stored albumin, and subsequently released a portion of it by encapsulating it in EVs during stimulation in a serum-free medium.

The co-presence of proteases and phosphatases in EVs suggests a complex bioactive machinery whose purpose appears to be both the modification of the tumor microenvironment and the modification of the basal state of the host cells with which they come into contact. In addition to LMW-PTPs, other enzymes could be contributing to the phosphatase activity observed on para-nitrophenol phosphate, such as alkaline phosphatase.

Our findings, derived from the in vitro model we used, are significantly reinforced by observations of histological sections of ovarian tissue using an inverted microscope, where we distinguished the presence of cells with protrusions similar in appearance to the large EVs we observed anchored to the surface of stimulated SKOV-3 cells. This was detected rarely and with difficulty in healthy ovaries, while it was easier to find several cells with these characteristics in samples of ovarian cancer diagnosed as serous papillary cystadenocarcinoma, suggesting that this phenomenon of large EV secretion (large oncosomes) occurs naturally in vivo, increasing in tumor tissue. This parallelism is vital, as it indicates that vesicles are not merely a cell culture artifact, but could play a role in the biology of ovarian cancer, possibly in tumor progression and dissemination. To date, few studies have focused on evaluating or reporting the possibility of observing large oncosomes in histological sections. One such study corresponds to prostate cancer, in which these large EVs were only observed in tumor tissue sections, and not in healthy tissue [38], and in lung tumor tissue [30].

This study provided a strategy for studying a wide variety of EVs in vitro, from small to very large, and thus we use the term PEVs. Although our study was limited to an ovarian cancer cell line, it opens the possibility of investigating the secretion behavior of PEVs by other ovarian cancer lines and other cancer types, using the bacterial fraction as an inducer, and thus conducting research studies in a context related to patients suffering from cancer and bacterial coinfections. In our working group, we will continue to investigate the induction of EV secretion from cancer cells in response to bacterial components, taking advantage of the bacterial fraction we have been using as a substrate, focusing on a larger number of cell lines for ovarian cancer and other types of cancer such as leukemia. We will also focus on further investigating the biological activity of EVs emanating from cancer cells, as well as their potential uses with prospects that benefit future patients.

## 4. Materials and Methods

### 4.1. Human Samples for Assays

All samples were taken during the hospital stay. The study was approved by the Ethics Committee of the Hospital Regional de Alta Especialidad de Ixtapaluca, “IMSS-Bienestar” (NR-017-2025).

### 4.2. Cell Culture

The SKOV-3 (ATCC-HTB-77) reference cell line was used. In brief, cell culture was performed in McCoy’s 5A medium (Corning, 10-050-CVR) at 37 °C and 5% CO_2_. Furthermore, for continuous maintenance of the medium, it was supplemented with 10% fetal bovine serum (FBS) (Corning, 35-010-CV) and 1% penicillin/streptomycin (PAA, P11-010), as described previously [46]. BFS was not used during polydisperse extracellular vesicle (PEV) secretion stimulations.

### 4.3. Extraction of Escherichia coli Fraction

Briefly, Gram-negative *E. coli* strain BL21(DE3) pLysS bacteria were cultured in 300 mL of Luria–Bertani (LB) medium, and a soluble bacterial fraction was obtained in 15 mL of 10 mM Tris -HCl pH 8.0, 10 mM MgCl_2_, and 2% SDS (fraction called SDS-SBMF). As described in the method modified by Sierra-López et al. 2025 [26], which was dialyzed in a cold room (temperature below 15 °C) using 15,000-Dalton membranes and 4 L of milliQ per sample, ending dialysis after 72 h.

### 4.4. Standardization of SKOV-3 PEV (Short and Large) Secretion and Isolation

In brief, glass coverslips or plastic surfaces coated with a mixture of 0.250 µg of human fibronectin (FN) and 20 ng of dialyzed SDS-SBMF protein per cm^2^ were used. The surfaces were prepared and the FN obtained as indicated by Sierra-López et al. 2025 [26]. No stimulant was placed as a negative control. SKOV-3 cells were deposited at a ratio of 4 × 10^4^/cm^2^ together with sufficient McCoy’s 5A medium with L-glutamine (Corning, 10–50-CV, USA) to coat the cultures without FBS. The cells were monitored under an inverted microscope, and after 120 min of interaction, the supernatant with the EVs was collected or fixed with 4% paraformaldehyde in PBS for 45 min for confocal microscopy assays. For SDS-PAGE, zymogram, and enzyme activity assays, EVs were isolated from cultures in 10 cm diameter Petri dishes containing 15 mL of initial medium, collecting 14 mL of supernatant after 2 h of interaction; the contaminating cells were then discarded together with 500 µL of contiguous supernatant by centrifuging at 120× *g* for 5 min in 15 mL tubes. The VE mix was precipitated with 0.2 mM ZnSO_4_ at 16,000× *g* for 20 min using 1.5 mL conical tubes (Centrifuge Eppendorf 5415C, EE.UU.).

### 4.5. SDS-PAGE and Western Blotting

The samples were resolved on 12% SDS-PAGE [47] and visualized by Coomassie blue or electrophoretically transferred to nitrocellulose membranes at 80 V. A total of 8 µL of β-mercaptoethanol, SDS-PAGE loading buffer, and cOmpleteTM protease inhibitor (Roche, Sigma-Aldrich) was added to each sample, which was then immediately boiled in a water bath for 8 min, followed by the addition of 8 µL of β-mercaptoethanol and basification using a 1M NaOH stock solution, as indicated in Sierra-López et al. 2021 [48]. Nitrocellulose Western blots (WBs) were incubated with mouse anti-human-LMW-PTP polyclonal antibodies at a dilution of 1:1000 (obtained from Sierra-López et al. 2025 [26]). Goat anti-mouse IgG (H + L) alkaline phosphatase conjugate (Invitrogen, Camarillo, CA, USA) at a dilution of 1:4000 in TBST was used as the secondary antibody. WBs were developed with the NBT/BCIP kit (Invitrogen, Camarillo, CA, USA).

### 4.6. Confocal Microscopy

SKOV-3 cells, either unstimulated or stimulated for 120 min with FN-SDS-SBMF on glass coverslips, were fixed with 4% p-formaldehyde in PBS for 45 min at 37 °C; they were gently washed with PBS and processed at room temperature. Permeabilization was performed with 0.0015% SDS and 0.0006% Triton X-100 in PBS for 8 min as indicated in Sierra-López et al. 2025 to reduce EV decoupling [26]. Samples were blocked with 5% BFS, washed with PBS, and incubated with anti-human-LMW-PTPs (1:300) in PBS for 1 h. They were then washed and incubated with FITC-conjugated anti-mouse as a secondary antibody (Zymed 1:300) for 1 h. Samples were preserved using Vectashield Antifade Reagent (Vector, Newark, CA, USA), examined with a Carl Zeiss LMS 700 confocal microscope, and processed with ZEN Blue 3.5 (Zeiss, White Plains, NY, USA). Green color was used to represent LMW-PTP loaded in EVs in the analysis with ImageJ2 (version 2.16.0) EVAnalyzer plugin (ImageJ/Fiji).

### 4.7. Liquid Chromatography and MALDI-MS/MS

Extracts from stimulated SKOV-3 cell EVs were resolved on 12% SDS-PAGE, and fragments between 10 and 200 kDa were processed at the Genomics, Proteomics, and Metabolomic Service of LaNSE (CINVESTAV IPN), according to the modified Shevchenko protocol [49], the method modified by Barrera-Rojas et al. 2018 [50] and as worked on by Sierra-López et al. 2025 [26]. The results of MS/MS spectra were compared using Protein Pilot v.2.0.1 (ABSciex, Framingham, MA, USA) and the Paragon algorithm [51] against *Homo sapiens* (Uniprot, 67,493 protein sequences database). The detection threshold was 1.3 to ensure 95% confidence. The identified proteins were grouped using the ProGroup algorithm to minimize redundancy. The profile and protein composition of the *E. coli* SDS-SBMF bacteria fraction were published in Sierra-López et al. 2025 [26].

### 4.8. Proteolytic Activity

Zymograms were performed to analyze the proteolytic activity of the EV mix using gelatin and fibronectin as substrates. EVs were prepared as previously described, avoiding heat denaturation and without β-ME, and were suspended with the standard loading buffer (glycerol, 0.5 M Tris-HCl, pH 6.8, SDS). Subsequently, 10 µg of EVs was separated by electrophoresis on standard 8% SDS–polyacrylamide gels (distilled water; acrylamide/bisacrylamide; 1.5 M Tris-HCl, pH 8.8; SDS; ammonium persulfate; and TEMED) copolymerized with 0.4% pork skin gelatin (Sigma G2500, Sigma-Aldrich, St. Louis, MO, USA) or 100 µg human fibronectin, and electrophoretic migration was performed at 4 °C without a reducing agent. The gels were washed with 1% Triton X-100 for 1 h, then incubated overnight at 37 °C in a developing buffer (50 mM Tris-HCl, 10 mM CaCl_2_, pH 7.0). The zymograms were stained with Coomassie blue; unstained areas indicated proteolytic activity.

### 4.9. Phosphatase Activity of Polydisperse Extracellular Vesicles (PEVs) of SKOV-3

The endpoint assay for phosphatase activity of SKOV-3 PVEs was performed using p-nitrophenyl phosphate (pNPP) (Sigma) as a substrate. A mixture was prepared with 60 µL of 200 mM sodium acetate pH 6.0 and 25 µL of 100 mM dithiothreitol (DTT). Approximately 5 µg of PEVs from samples obtained from stimulated supernatants without using ZnSO4 (to avoid interference) was added. MilliQ water was added to a total volume of 120 µL, followed by incubation for 10 min at room temperature. The mixture was then incubated with 2 µL of 100 mM pNPP and mixed by vortexing for 1 min. The mixture was then incubated at 37 °C. A total of 100 µL of reaction solution was taken after 2 h, and the reaction was stopped with 10 µL of 2 M NaOH to read the absorbance at 405 nm in 96-well plates. The PEV samples were added without breaking them or in the extract after 1 min of sonication in an ice bath. We detected that LMW-PTP was degraded in PEV extracts, so the phosphatase enzyme activity of the unlysed PEVs was tested.

### 4.10. Statistical Analysis

Confocal analysis of PEV secretion by SKOV-3 cells was performed using Fiji and the EV Analyzer plugin (version 8.1.3 beta). Function: PEV count; identical thresholds: 10; threshold method: ‘Li’; min circularity: 0.5; filter type: PEV-GFP (to FITC channel); particle size range: 1–999,999. Data was analyzed using GraphPad Prism 5 Software: One-way Anova Bonferroni post-test.

## 5. Conclusions

In summary, our findings suggest that the outer membrane fraction of the Gram-negative bacteria *E. coli* is a potent inductor of polydisperse extracellular vesicle (PEV) secretion in the SKOV-3 ovarian cancer cell line, a mechanism potentially amplified by the synergistic effect of bacterial components like LPS and porins. The PEVs function as protective compartments that encapsulate and maintain a complex bioactive machinery of proteases and phosphatases (including LMW-PTP), which, upon release, actively modify the tumor microenvironment by degrading the extracellular matrix (e.g., fibronectin). This modification correlates with an elongated and potentially more migratory cellular phenotype. The detection of structures resembling large PEVs (large oncosomes) in ovarian cancer tissue, in contrast to healthy tissue, critically validates the in vivo relevance of this phenomenon. However, it must be noted that these findings are currently limited to the SKOV-3 cell line. Collectively, these data suggest that bacterial coinfections can exacerbate cancer progression and dissemination by robustly stimulating PEV secretion, underscoring the importance of this communication axis in tumor biology and opening avenues for investigating other cancer types.

## Data Availability

The original contributions presented in this study are included in the article/supplementary material. Further inquiries can be directed to the corresponding authors.

## Supplementary Materials

The following supporting information can be downloaded at https://www.mdpi.com/article/10.3390/ijms262110653/s1.

## Abbreviations

EVs	Extracellular vesicles
PEVs	Polydisperse extracellular vesicles
LPS	Lipopolysaccharide
SKOV-3	Human ovarian cancer cell line
SBMF-SDS	SDS-soluble bacterial membrane fraction
LMW-PTP	Low-molecular-weight protein tyrosine phosphatase
MALDI-MS/MS	Matrix-assisted laser desorption/ionization mass spectrometry
OC	Ovarian cancer
FN	Fibronectin
Omp	Outer membrane protein
