# Supplementary material for: Secretion of Extracellular Microvesicles Induced by a Fraction of Escherichia coli: Possible Role in Ovarian Cancer with Bacterial Coinfections
Source: Int J Mol Sci. 2025 Nov 1;26(21):10653. doi: 10.3390/ijms262110653 (PMC12608175; doi:10.3390/ijms262110653)
Supplement: Supplementary file 1 [file ijms-26-10653-s001.zip › ijms-3907383-supplementary.pdf]

Supplementary

Table S1. Proteins in the *E. coli* fraction that was used to stimulated SKOV-3 ovarian cancer EVs secretion was previously identified (Sierra-López et al, 2025) using: U: Unused > 1.3 = 95% confidence. Significant peptide (95%). MALDI TOF TOF was used. Frag: SDS-PAGE Fragment used to the analysis.

Frag	U	Accession	Name	Peptides (95%)
F1	12.16	sp|P0CE48|EFTU2_ECOLI	Elongation factor Tu 2 OS=Escherichia coli (strain K12) GN=tufB PE=1 SV=1	8
	5.28	sp|P0ABB4|ATPB_ECOLI	ATP synthase subunit beta OS=Escherichia coli (strain K12) GN=atpD PE=1 SV=2	3
	4.74	sp|P02931|OMPF_ECOLI	Outer membrane protein F OS=Escherichia coli (strain K12) GN=ompF PE=1 SV=1	2
	4.08	sp|P31554|LPTD_ECOLI	LPS-assembly protein LptD OS=Escherichia coli (strain K12) GN=lptD PE=1 SV=2	2
	4	sp|P0AG67|RS1_ECOLI	30S ribosomal protein S1 OS=Escherichia coli (strain K12) GN=rpsA PE=1 SV=1	2
	2.28	sp|P0ABJ9|CYDA_ECOLI	Cytochrome bd-I ubiquinol oxidase subunit 1 OS=Escherichia coli (strain K12) GN=cydA PE=1 SV=1	1
F2	11.81	sp|P0CE48|EFTU2_ECOLI	Elongation factor Tu 2 OS=Escherichia coli (strain K12) GN=tufB PE=1 SV=1	9
	10	sp|P02931|OMPF_ECOLI	Outer membrane protein F OS=Escherichia coli (strain K12) GN=ompF PE=1 SV=1	6
	8.41	sp|P0AG67|RS1_ECOLI	30S ribosomal protein S1 OS=Escherichia coli (strain K12) GN=rpsA PE=1 SV=1	4
	8	sp|P0ABB4|ATPB_ECOLI	ATP synthase subunit beta OS=Escherichia coli (strain K12) GN=atpD PE=1 SV=2	4
	8	sp|P0A836|SUCC_ECOLI	Succinyl-CoA ligase [ADP-forming] subunit beta OS=Escherichia coli (strain K12) GN=sucC PE=1 SV=1	6
	6.52	sp|P0A940|BAMA_ECOLI	Outer membrane protein assembly factor BamA OS=Escherichia coli (strain K12) GN=bamA PE=1 SV=1	3
	6.15	sp|P0ABJ9|CYDA_ECOLI	Cytochrome bd-I ubiquinol oxidase subunit 1 OS=Escherichia coli (strain K12) GN=cydA PE=1 SV=1	3
	4.19	sp|P0AAI3|FTSH_ECOLI	ATP-dependent zinc metalloprotease FtsH OS=Escherichia coli (strain K12) GN=ftsH PE=1 SV=1	2
	4.06	sp|P33136|OPGG_ECOLI	Glucans biosynthesis protein G OS=Escherichia coli (strain K12) GN=mdoG PE=1 SV=1	2
	2.02	sp|P0AC41|SDHA_ECOLI	Succinate dehydrogenase flavoprotein subunit OS=Escherichia coli (strain K12) GN=sdhA PE=1 SV=1	2
	2	sp|P02919|PBPB_ECOLI	Penicillin-binding protein 1B OS=Escherichia coli (strain K12) GN=mrcB PE=1 SV=2	1
	2	sp|P33599|NUOCD_ECOLI	NADH-quinone oxidoreductase subunit C/D OS=Escherichia coli (strain K12) GN=nuoC PE=1 SV=3	1
	2	sp|P0ABB0|ATPA_ECOLI	ATP synthase subunit alpha OS=Escherichia coli (strain K12) GN=atpA PE=1 SV=1	1
	2	sp|P0A825|GLYA_ECOLI	Serine hydroxymethyltransferase OS=Escherichia coli (strain K12) GN=glyA PE=1 SV=1	1
	2	sp|P0A6F5|CH60_ECOLI	60 kDa chaperonin OS=Escherichia coli (strain K12) GN=groL PE=1 SV=2	2
	1.59	sp|P25526|GABD_ECOLI	Succinate-semialdehyde dehydrogenase [NADP(+)] GabD OS=Escherichia coli (strain K12) GN=gabD PE=1 SV=1	1
	1.35	sp|P0CB39|EPTC_ECOLI	Phosphoethanolamine transferase EptC OS=Escherichia coli (strain K12) GN=eptC PE=1 SV=1	1
F3	36.07	sp|P0ABB4|ATPB_ECOLI	ATP synthase subunit beta OS=Escherichia coli (strain K12) GN=atpD PE=1 SV=2	24

	30	sp|P0ABB0|ATPA_ECOLI	ATP synthase subunit alpha OS=Escherichia coli (strain K12) GN=atpA PE=1 SV=1	19
	28.28	sp|P02930|TOLC_ECOLI	Outer membrane protein TolC OS=Escherichia coli (strain K12) GN=tolC PE=1 SV=3	22
	27.52	sp|P02943|LAMB_ECOLI	Maltoporin OS=Escherichia coli (strain K12) GN=lamB PE=1 SV=1	33
	12.05	sp|P0ABJ9|CYDA_ECOLI	Cytochrome bd-I ubiquinol oxidase subunit 1 OS=Escherichia coli (strain K12) GN=cydA PE=1 SV=1	7
	10.03	sp|P0CE48|EFTU2_ECOLI	Elongation factor Tu 2 OS=Escherichia coli (strain K12) GN=tufB PE=1 SV=1	10
	0	sp|P0CE47|EFTU1_ECOLI	Elongation factor Tu 1 OS=Escherichia coli (strain K12) GN=tufA PE=1 SV=1	10
	6	sp|P02931|OMPF_ECOLI	Outer membrane protein F OS=Escherichia coli (strain K12) GN=ompF PE=1 SV=1	3
	4	sp|P0ABC7|HFLK_ECOLI	Modulator of FtsH protease HflK OS=Escherichia coli (strain K12) GN=hflK PE=1 SV=1	2
	2.01	sp|P68187|MALK_ECOLI	Maltose/maltodextrin import ATP-binding protein MalK OS=Escherichia coli (strain K12) GN=malK PE=1 SV=1	1
	2	sp|P28903|NRDD_ECOLI	Anaerobic ribonucleoside-triphosphate reductase OS=Escherichia coli (strain K12) GN=nrdD PE=1 SV=2	1
	2	sp|Q46814|XDHD_ECOLI	Probable hypoxanthine oxidase XdhD OS=Escherichia coli (strain K12) GN=xdhD PE=3 SV=1	1
	2	sp|P00393|DHNA_ECOLI	NADH dehydrogenase OS=Escherichia coli (strain K12) GN=ndh PE=1 SV=2	1
	2	sp|P60872|YIDE_ECOLI	Putative transport protein YidE OS=Escherichia coli (strain K12) GN=yidE PE=3 SV=1	1
	1.51	sp|P0AA78|EXUT_ECOLI	Hexuronate transporter OS=Escherichia coli (strain K12) GN=exuT PE=1 SV=2	1
F4	26.72	sp|P02931|OMPF_ECOLI	Outer membrane protein F OS=Escherichia coli (strain K12) GN=ompF PE=1 SV=1	40
F5	20	sp|P0A910|OMPA_ECOLI	Outer membrane protein A OS=Escherichia coli (strain K12) GN=ompA PE=1 SV=1	13
	8	sp|P02931|OMPF_ECOLI	Outer membrane protein F OS=Escherichia coli (strain K12) GN=ompF PE=1 SV=1	4
F6	12.13	sp|P02931|OMPF_ECOLI	Outer membrane protein F OS=Escherichia coli (strain K12) GN=ompF PE=1 SV=1	8
	10	sp|P0A910|OMPA_ECOLI	Outer membrane protein A OS=Escherichia coli (strain K12) GN=ompA PE=1 SV=1	5
	4.77	sp|P0A927|TSX_ECOLI	Nucleoside-specific channel-forming protein tsx OS=Escherichia coli (strain K12) GN=tsx PE=1 SV=1	3
	2	sp|P0A7L0|RL1_ECOLI	50S ribosomal protein L1 OS=Escherichia coli (strain K12) GN=rplA PE=1 SV=2	1
	2	sp|P0ABJ9|CYDA_ECOLI	Cytochrome bd-I ubiquinol oxidase subunit 1 OS=Escherichia coli (strain K12) GN=cydA PE=1 SV=1	1
F7	6.13	sp|P02931|OMPF_ECOLI	Outer membrane protein F OS=Escherichia coli (strain K12) GN=ompF PE=1 SV=1	3
	3.85	sp|P0AG55|RL6_ECOLI	50S ribosomal protein L6 OS=Escherichia coli (strain K12) GN=rplF PE=1 SV=2	2
	2.77	sp|P62399|RL5_ECOLI	50S ribosomal protein L5 OS=Escherichia coli (strain K12) GN=rplE PE=1 SV=2	1
	1.5	sp|P0ABJ9|CYDA_ECOLI	Cytochrome bd-I ubiquinol oxidase subunit 1 OS=Escherichia coli (strain K12) GN=cydA PE=1 SV=1	1
F8	16.04	sp|P0A917|OMPX_ECOLI	Outer membrane protein X OS=Escherichia coli (strain K12) GN=ompX PE=1 SV=1	8
	4	sp|P69776|LPP_ECOLI	Major outer membrane lipoprotein Lpp OS=Escherichia coli (strain K12) GN=lpp PE=1 SV=1	2
	2.01	sp|P69411|RCSF_ECOLI	Outer membrane lipoprotein RcsF OS=Escherichia coli (strain K12) GN=rcsF PE=1 SV=1	1
	2	sp|P0ADY7|RL16_ECOLI	50S ribosomal protein L16 OS=Escherichia coli (strain K12) GN=rplP PE=1 SV=1	1

	2	sp|P0AEU7|SKP_ECOLI	Chaperone protein Skp OS=Escherichia coli (strain K12) GN=skp PE=1 SV=1	2
	2	sp|P0A7J3|RL10_ECOLI	50S ribosomal protein L10 OS=Escherichia coli (strain K12) GN=rplJ PE=1 SV=2	1

F9	12	sp|P0ADW3|YHCB_ECOLI	Inner membrane protein YhcB OS=Escherichia coli (strain K12) GN=yhcB PE=1 SV=2	7
	12	sp|P02931|OMPF_ECOLI	Outer membrane protein F OS=Escherichia coli (strain K12) GN=ompF PE=1 SV=1	9
	10	sp|P68919|RL25_ECOLI	50S ribosomal protein L25 OS=Escherichia coli (strain K12) GN=rplY PE=1 SV=1	5
	8.01	sp|P0A905|SLYB_ECOLI	Outer membrane lipoprotein SlyB OS=Escherichia coli (strain K12) GN=slyB PE=2 SV=1	9
	8	sp|P61175|RL22_ECOLI	50S ribosomal protein L22 OS=Escherichia coli (strain K12) GN=rplV PE=1 SV=1	6
	8	sp|P0A7K6|RL19_ECOLI	50S ribosomal protein L19 OS=Escherichia coli (strain K12) GN=rplS PE=1 SV=2	4
	6.06	sp|P0A7R5|RS10_ECOLI	30S ribosomal protein S10 OS=Escherichia coli (strain K12) GN=rpsJ PE=1 SV=1	3
	6.01	sp|P0A910|OMPA_ECOLI	Outer membrane protein A OS=Escherichia coli (strain K12) GN=ompA PE=1 SV=1	3
	6.01	sp|P0A7S9|RS13_ECOLI	30S ribosomal protein S13 OS=Escherichia coli (strain K12) GN=rpsM PE=1 SV=2	3
	4	sp|P0A7R9|RS11_ECOLI	30S ribosomal protein S11 OS=Escherichia coli (strain K12) GN=rpsK PE=1 SV=2	2
	4	sp|P0AG48|RL21_ECOLI	50S ribosomal protein L21 OS=Escherichia coli (strain K12) GN=rplU PE=1 SV=1	3
	4	sp|P0A7J7|RL11_ECOLI	50S ribosomal protein L11 OS=Escherichia coli (strain K12) GN=rplK PE=1 SV=2	2
	4	sp|P0A7T7|RS18_ECOLI	30S ribosomal protein S18 OS=Escherichia coli (strain K12) GN=rpsR PE=1 SV=2	2
	4	sp|P0C054|IBPA_ECOLI	Small heat shock protein IbpA OS=Escherichia coli (strain K12) GN=ibpA PE=1 SV=1	2
	4	sp|P0AG44|RL17_ECOLI	50S ribosomal protein L17 OS=Escherichia coli (strain K12) GN=rplQ PE=1 SV=1	2
	4	sp|P0A7U3|RS19_ECOLI	30S ribosomal protein S19 OS=Escherichia coli (strain K12) GN=rpsS PE=1 SV=2	2
	4	sp|P0ADZ7|YAJC_ECOLI	UPF0092 membrane protein YajC OS=Escherichia coli (strain K12) GN=yajC PE=1 SV=1	3
	4	sp|P0A917|OMPX_ECOLI	Outer membrane protein X OS=Escherichia coli (strain K12) GN=ompX PE=1 SV=1	2
	4	sp|P69776|LPP_ECOLI	Major outer membrane lipoprotein Lpp OS=Escherichia coli (strain K12) GN=lpp PE=1 SV=1	5
	4	sp|P60624|RL24_ECOLI	50S ribosomal protein L24 OS=Escherichia coli (strain K12) GN=rplX PE=1 SV=2	2
	2.16	sp|P68679|RS21_ECOLI	30S ribosomal protein S21 OS=Escherichia coli (strain K12) GN=rpsU PE=1 SV=2	1
	2.05	sp|P0ADY3|RL14_ECOLI	50S ribosomal protein L14 OS=Escherichia coli (strain K12) GN=rplN PE=1 SV=1	1
	2.04	sp|P0A7U7|RS20_ECOLI	30S ribosomal protein S20 OS=Escherichia coli (strain K12) GN=rpsT PE=1 SV=2	1
	2.03	sp|P0A7R1|RL9_ECOLI	50S ribosomal protein L9 OS=Escherichia coli (strain K12) GN=rplI PE=1 SV=1	1
	2.01	sp|P0A7L8|RL27_ECOLI	50S ribosomal protein L27 OS=Escherichia coli (strain K12) GN=rpmA PE=1 SV=2	1
	2	sp|P0C018|RL18_ECOLI	50S ribosomal protein L18 OS=Escherichia coli (strain K12) GN=rplR PE=1 SV=1	1
	2	sp|P06715|GSHR_ECOLI	Glutathione reductase OS=Escherichia coli (strain K12) GN=gor PE=1 SV=1	1
	1.34	sp|P0ACF0|DBHA_ECOLI	DNA-binding protein HU-alpha OS=Escherichia coli (strain K12) GN=hupA PE=1 SV=1	1

Table S2. Secretion of EVs secreted by SKOV-3 ovarian cancer cells were analyzes by Fiji and the EVAnalyzer plujing (version 8.1.3 beta). Identical thresholds 10, Thershold method 'Li', Min circularity: 0.5, Filter Type EV-GFP and identical settings for all conditions that are compared were used on confocal images with FITC fluorescence channel (anti-LMW-PTP). AU: Arbitrary units. Valid: number of EVs detected. The table is repre-sentative of the amount of EVs near and/or anchored per each SKOV-3 cell cytoplasmic membrane surface. In unstimulated SKOV-3 ovarian cancer cells, the cells that secrete EVs were sought.

Stimulated SKOV-3 EVs_FITC				
area size (AU)	intensity	circularity [0-1]	valid	invalid
9.00	16.89	1.00	1.00	0.00
0.00	0.00	0.00	0.00	0.00
20.40	15.87	0.91	10.00	8.00
14.22	15.28	0.87	9.00	9.00
0.00	0.00	0.00	0.00	0.00
16.71	14.31	0.93	7.00	7.00
Unstimulated SKOV-3 EVs_FITC				
4.00	17.25	0.86	1.00	1.00
0.00	0.00	0.00	0.00	0.00
0.00	0.00	0.00	0.00	0.00
0.00	0.00	0.00	0.00	0.00
0.00	0.00	0.00	0.00	0.00
0.00	0.00	0.00	0.00	0.00

Table S3. Protein identification in the mix of EVs (short and large) released by stimulated SKOV-3 ovarian cancer. U: Unused > 1.3 = 95 % confidence. Significant peptide (95%). MALDI TOF TOF was used. Frag: SDS-PAGE Fragment used to the analysis.

Frag	U	Accession	Name	Peptides 95%
F1	30.56	sp|P21333|FLNA	Filamin-A OS=Homo sapiens GN=FLNA PE=1 SV=4	13
	8.15	sp|O75369|FLNB	Filamin-B OS=Homo sapiens GN=FLNB PE=1 SV=2	4
	7.93	sp|P63261|ACTG	Actin, cytoplasmic 2 OS=Homo sapiens GN=ACTG1 PE=1 SV=1	5
	7.93	sp|P60709|ACTB	Actin, cytoplasmic 1 OS=Homo sapiens GN=ACTB PE=1 SV=1	5
	7.37	sp|P02768|ALBU	Serum albumin OS=Homo sapiens GN=ALB PE=1 SV=2	4
	6.76	sp|Q9Y490|TLN1	Talin-1 OS=Homo sapiens GN=TLN1 PE=1 SV=3	4
	6.48	sp|Q00610|CLH1	Clathrin heavy chain 1 OS=Homo sapiens GN=CLTC PE=1 SV=5	3
	6.48	sp|Q00610-2|CLH1	Isoform 2 of Clathrin heavy chain 1 OS=Homo sapiens GN=CLTC	3
	6.17	sp|P68363|TBA1B	Tubulin alpha-1B chain OS=Homo sapiens GN=TUBA1B PE=1 SV=1	3
	6.17	sp|P68363-2|TBA1B	Isoform 2 of Tubulin alpha-1B chain OS=Homo sapiens GN=TUBA1B	3
	5.27	sp|P02751|FINC	Fibronectin OS=Homo sapiens GN=FN1 PE=1 SV=4	2
	4.47	sp|P07437|TBB5	Tubulin beta chain OS=Homo sapiens GN=TUBB PE=1 SV=2	2
	4.09	sp|P01023|A2MG	Alpha-2-macroglobulin OS=Homo sapiens GN=A2M PE=1 SV=3	2
	4.05	sp|P15311|EZRI	Ezrin OS=Homo sapiens GN=EZR PE=1 SV=4	2

	4	sp|P35241|RADI	Radixin OS=Homo sapiens GN=RDX PE=1 SV=1	2
	2.55	sp|P14618|KPYM	Pyruvate kinase PKM OS=Homo sapiens GN=PKM PE=1 SV=4	1
	2.5	sp|P14618-2|KPYM	Isoform M1 of Pyruvate kinase PKM OS=Homo sapiens GN=PKM	1
	2.19	sp|P02787|TRFE	Serotransferrin OS=Homo sapiens GN=TF PE=1 SV=3	1
	2	tr|E7ER44|E7ER44	Lactotransferrin OS=Homo sapiens GN=LTF PE=1 SV=1	1
	2	tr|Q5T985|Q5T985	Inter-alpha-trypsin inhibitor heavy chain H2 OS=Homo sapiens GN=ITIH2 PE=1 SV=1	1
	2	tr|F8W6P5|F8W6P5	LVV-hemorphin-7 (Fragment) OS=Homo sapiens GN=HBB PE=1 SV=1	1
F2	10	tr|B7WNR0|B7WNR0	Serum albumin OS=Homo sapiens GN=ALB PE=1 SV=1	5
	8.27	sp|P07900|HS90A	Heat shock protein HSP 90-alpha OS=Homo sapiens GN=HSP90AA1 PE=1 SV=5	5
	8.27	sp|P07900-2|HS90A	Isoform 2 of Heat shock protein HSP 90-alpha OS=Homo sapiens GN=HSP90AA1	5
	6.13	sp|O43707|ACTN4	Alpha-actinin-4 OS=Homo sapiens GN=ACTN4 PE=1 SV=2	3
	6.13	sp|O43707-2|ACTN4	Isoform ACTN4ISO of Alpha-actinin-4 OS=Homo sapiens GN=ACTN4	3
	6.06	sp|P63261|ACTG	Actin, cytoplasmic 2 OS=Homo sapiens GN=ACTG1 PE=1 SV=1	3
	6.06	sp|P60709|ACTB	Actin, cytoplasmic 1 OS=Homo sapiens GN=ACTB PE=1 SV=1	3
	4.03	sp|P19338|NUCL	Nucleolin OS=Homo sapiens GN=NCL PE=1 SV=3	2
	4.02	sp|Q9BQE3|TBA1C	Tubulin alpha-1C chain OS=Homo sapiens GN=TUBA1C PE=1 SV=1	2
	4.02	sp|Q71U36|TBA1A	Tubulin alpha-1A chain OS=Homo sapiens GN=TUBA1A PE=1 SV=1	2
	4.02	sp|Q71U36-2|TBA1A	Isoform 2 of Tubulin alpha-1A chain OS=Homo sapiens GN=TUBA1A	2
	2.03	sp|Q8WZ42-12|TITIN	Isoform 12 of Titin OS=Homo sapiens GN=TTN	1
	2.03	sp|Q8WZ42|TITIN	Titin OS=Homo sapiens GN=TTN PE=1 SV=4 (e isoformas 2-11 excepto 6)	1
	2	sp|P12814-4|ACTN1	Isoform 4 of Alpha-actinin-1 OS=Homo sapiens GN=ACTN1	2
	2	sp|P12814|ACTN1	Alpha-actinin-1 OS=Homo sapiens GN=ACTN1 PE=1 SV=2	2
	2	sp|P26038|MOES	Moesin OS=Homo sapiens GN=MSN PE=1 SV=3	1
	2	sp|P35241|RADI	Radixin OS=Homo sapiens GN=RDX PE=1 SV=1	1
	2	sp|Q12906|ILF3	Interleukin enhancer-binding factor 3 OS=Homo sapiens GN=ILF3 PE=1 SV=3	1
	2	sp|Q12906-7|ILF3	Isoform 7 of Interleukin enhancer-binding factor 3 OS=Homo sapiens GN=ILF3	1
	2	sp|O94819|KBTBB	Kelch repeat and BTB domain-containing protein 11 OS=Homo sapiens GN=KBTBD11 PE=1 SV=1	1
	2	tr|H3BS10|H3BS10	Beta-hexosaminidase OS=Homo sapiens GN=HEXA PE=1 SV=1	1
F3	17.87	sp|P11142|HSP7C	Heat shock cognate 71 kDa protein OS=Homo sapiens GN=HSPA8 PE=1 SV=1	17
	17.21	sp|P15311|EZRI	Ezrin OS=Homo sapiens GN=EZR PE=1 SV=4	7
	14.28	sp|P08107|HSP71	Heat shock 70 kDa protein 1A/1B OS=Homo sapiens GN=HSPA1A PE=1 SV=5	10
	9.27	sp|P14618|KPYM	Pyruvate kinase PKM OS=Homo sapiens GN=PKM PE=1 SV=4	9
	7.48	sp|P14618-2|KPYM	Isoform M1 of Pyruvate kinase PKM OS=Homo sapiens GN=PKM	8
	7.41	sp|P26038|MOES	Moesin OS=Homo sapiens GN=MSN PE=1 SV=3	8
	5.34	sp|P11021|GRP78	78 kDa glucose-regulated protein OS=Homo sapiens GN=HSPA5 PE=1 SV=2	3
	4.3	sp|P02768|ALBU	Serum albumin OS=Homo sapiens GN=ALB PE=1 SV=2	6
	3.44	tr|F5H5D3|F5H5D3	Tubulin alpha-1C chain OS=Homo sapiens GN=TUBA1C PE=1 SV=1	2

	3.25	sp|P29401|TKT	Transketolase OS=Homo sapiens GN=TKT PE=1 SV=3	2
	3.16	tr|E7ER44|E7ER44	Lactotransferrin OS=Homo sapiens GN=LTF PE=1 SV=1	7
	1.29	sp|P02787|TRFE_HUMAN	Serotransferrin OS=Homo sapiens GN=TF PE=1 SV=3	8
	3.16	sp|P02788-2|TRFL	Isoform DeltaLf of Lactotransferrin OS=Homo sapiens GN=LTF	7
	2.94	sp|P61978|HNRPK	Heterogeneous nuclear ribonucleoprotein K OS=Homo sapiens GN=HNRNPK PE=1 SV=1	2
	2.47	sp|P10809|CH60	60 kDa heat shock protein, mitochondrial OS=Homo sapiens GN=HSPD1 PE=1 SV=2	2
F4	11.39	sp|P08670|VIME	Vimentin OS=Homo sapiens GN=VIM PE=1 SV=4	6
	7.96	sp|P02768|ALBU	Serum albumin OS=Homo sapiens GN=ALB PE=1 SV=2	13
	5.86	tr|F5H5D3|F5H5D3	Tubulin alpha-1C chain OS=Homo sapiens GN=TUBA1C PE=1 SV=1	3
	5.86	sp|Q71U36|TBA1A	Tubulin alpha-1A chain OS=Homo sapiens GN=TUBA1A PE=1 SV=1	3
	5.29	sp|P30101|PDIA3	Protein disulfide-isomerase A3 OS=Homo sapiens GN=PDIA3 PE=1 SV=4	2
	2.51	sp|P14618|KPYM	Pyruvate kinase PKM OS=Homo sapiens GN=PKM PE=1 SV=4	1
	2	sp|P14618-2|KPYM	Isoform M1 of Pyruvate kinase PKM OS=Homo sapiens GN=PKM	1
	2.15	sp|Q01518|CAP1	Adenylyl cyclase-associated protein 1 OS=Homo sapiens GN=CAP1 PE=1 SV=5	1
	2.15	sp|Q01518-2|CAP1	Isoform 2 of Adenylyl cyclase-associated protein 1 OS=Homo sapiens GN=CAP1	1
	2.11	sp|P63261|ACTG	Actin, cytoplasmic 2 OS=Homo sapiens GN=ACTG1 PE=1 SV=1	1
	2.11	sp|P60709|ACTB	Actin, cytoplasmic 1 OS=Homo sapiens GN=ACTB PE=1 SV=1	1
	2	sp|P06744-2|G6PI	Isoform 2 of Glucose-6-phosphate isomerase OS=Homo sapiens GN=GPI	1
	2	sp|P06744|G6PI	Glucose-6-phosphate isomerase OS=Homo sapiens GN=GPI PE=1 SV=4	1
	2	sp|P11413-3|G6PD	Isoform 3 of Glucose-6-phosphate 1-dehydrogenase OS=Homo sapiens GN=G6PD	1
	2	sp|Q8NC51|PAIRB	Plasminogen activator inhibitor 1 RNA-binding protein OS=Homo sapiens GN=SERBP1 PE=1 SV=2	1
	1.45	tr|Q5JP53|Q5JP53	Tubulin beta chain OS=Homo sapiens GN=TUBB PE=1 SV=1	1
F5	20.47	sp|P06733|ENOA	Alpha-enolase OS=Homo sapiens GN=ENO1 PE=1 SV=2	15
	16.67	sp|P07437|TBB5	Tubulin beta chain OS=Homo sapiens GN=TUBB PE=1 SV=2	8
	15.88	sp|P63261|ACTG	Actin, cytoplasmic 2 OS=Homo sapiens GN=ACTG1 PE=1 SV=1	10
	14	sp|P68104|EF1A1	Elongation factor 1-alpha 1 OS=Homo sapiens GN=EEF1A1 PE=1 SV=1	8
	12.04	sp|P00558|PGK1	Phosphoglycerate kinase 1 OS=Homo sapiens GN=PGK1 PE=1 SV=3	6
	12.04	sp|P00558-2|PGK1	Isoform 2 of Phosphoglycerate kinase 1 OS=Homo sapiens GN=PGK1	6
	8.4	sp|P26641|EF1G	Elongation factor 1-gamma OS=Homo sapiens GN=EEF1G PE=1 SV=3	4
	6.35	sp|P26641-2|EF1G	Isoform 2 of Elongation factor 1-gamma OS=Homo sapiens GN=EEF1G	3
	8	tr|Q5STU3|Q5STU3	Spliceosome RNA helicase DDX39B OS=Homo sapiens GN=DDX39B PE=1 SV=2	4
	6.79	sp|P68363|TBA1B	Tubulin alpha-1B chain OS=Homo sapiens GN=TUBA1B PE=1 SV=1	4
	6	sp|Q06830|PRDX1	Peroxiredoxin-1 OS=Homo sapiens GN=PRDX1 PE=1 SV=1	3
	4.24	sp|P02768|ALBU	Serum albumin OS=Homo sapiens GN=ALB PE=1 SV=2	3
	4.15	sp|P60842|IF4A1	Eukaryotic initiation factor 4A-I OS=Homo sapiens	2

			GN=EIF4A1 PE=1 SV=1	
	4.15	sp|P60842-2|IF4A1	Isoform 2 of Eukaryotic initiation factor 4A-I OS=Homo sapiens GN=EIF4A1	2
	2.72	sp|P23526|SAHH	Adenosylhomocysteinase OS=Homo sapiens GN=AHCY PE=1 SV=4	1
	2.4	tr|G3V4C1|G3V4C1	Heterogeneous nuclear ribonucleoproteins C1/C2 OS=Homo sapiens GN=HNRNPC PE=1 SV=1	1
	2.22	sp|P50502|F10A1	Hsc70-interacting protein OS=Homo sapiens GN=ST13 PE=1 SV=2	1
	2.12	tr|F8VZY9|F8VZY9	Keratin, type I cytoskeletal 18 OS=Homo sapiens GN=KRT18 PE=1 SV=1	1
	2.12	sp|P40121|CAPG	Macrophage-capping protein OS=Homo sapiens GN=CAPG PE=1 SV=2	1
	2.12	sp|P40121-2|CAPG	Isoform 2 of Macrophage-capping protein OS=Homo sapiens GN=CAPG	1
	2.04	tr|A0A087X027| A0A087X027	Protein SETSIP OS=Homo sapiens GN=SETSIP PE=4 SV=1	1
	2.03	sp|P04406|G3P	Glyceraldehyde-3-phosphate dehydrogenase OS=Homo sapiens GN=GAPDH PE=1 SV=3	1
	2	sp|P68133|ACTS	Actin, alpha skeletal muscle OS=Homo sapiens GN=ACTA1 PE=1 SV=1	6
	2	tr|J3KPG5|J3KPG5	Bromodomain adjacent to zinc finger domain protein 2A OS=Homo sapiens GN=BAZ2A PE=1 SV=1	1
	2	sp|P07355|ANXA2	Annexin A2 OS=Homo sapiens GN=ANXA2 PE=1 SV=2	1
	2	sp|P67809|YBOX1	Nuclease-sensitive element-binding protein 1 OS=Homo sapiens GN=YBX1 PE=1 SV=3	1
	2	tr|H0Y8G5|H0Y8G5	Heterogeneous nuclear ribonucleoprotein D0 (Fragment) OS=Homo sapiens GN=HNRNPD PE=1 SV=3	1
	2	sp|P00966|ASSY	Argininosuccinate synthase OS=Homo sapiens GN=ASS1 PE=1 SV=2	1
	2	sp|O95302-3|FKBP9	Isoform 3 of Peptidyl-prolyl cis-trans isomerase FKBP9 OS=Homo sapiens GN=FKBP9	1
	2	sp|O95302|FKBP9	Peptidyl-prolyl cis-trans isomerase FKBP9 OS=Homo sapiens GN=FKBP9 PE=1 SV=2	1
	2	tr|J3KPS3|J3KPS3	Fructose-bisphosphate aldolase OS=Homo sapiens GN=ALDOA PE=1 SV=1	1
	2	sp|P55795|HNRH2	Heterogeneous nuclear ribonucleoprotein H2 OS=Homo sapiens GN=HNRNPH2 PE=1 SV=1	1
	2	sp|P39748|FEN1	Flap endonuclease 1 OS=Homo sapiens GN=FEN1 PE=1 SV=1	1
	2	sp|P02774-3|VTDB	Isoform 3 of Vitamin D-binding protein OS=Homo sapiens GN=GC	1
F6	12.87	sp|P07355|ANXA2	Annexin A2 OS=Homo sapiens GN=ANXA2 PE=1 SV=2	9
	12.87	sp|P07355-2|ANXA2	Isoform 2 of Annexin A2 OS=Homo sapiens GN=ANXA2	9
	9.88	tr|Q6P452|Q6P452	Annexin A4 OS=Homo sapiens GN=ANXA4 PE=1 SV=1	5
	7.66	sp|P04406|G3P	Glyceraldehyde-3-phosphate dehydrogenase OS=Homo sapiens GN=GAPDH PE=1 SV=3	4
	6.3	sp|P12429|ANXA3	Annexin A3 OS=Homo sapiens GN=ANXA3 PE=1 SV=3	3
	6.06	sp|P08758|ANXA5	Annexin A5 OS=Homo sapiens GN=ANXA5 PE=1 SV=2	3
	5.7	sp|P00338|LDHA	L-lactate dehydrogenase A chain OS=Homo sapiens GN=LDHA PE=1 SV=2	2
	4.44	sp|P04083|ANXA1	Annexin A1 OS=Homo sapiens GN=ANXA1 PE=1 SV=2	7
	3.58	sp|Q06830|PRDX1	Peroxiredoxin-1 OS=Homo sapiens GN=PRDX1 PE=1 SV=1	3
	2.87	sp|E9PAV3|NACAM	Nascent polypeptide-associated complex subunit alpha, muscle-specific form OS=Homo sapiens GN=NACA PE=1 SV=1	1
	2.81	sp|P22626|ROA2	Heterogeneous nuclear ribonucleoproteins A2/B1 OS=Homo sapiens GN=HNRNPA2B1 PE=1 SV=2	3
	2.31	sp|Q14847|LASP1	LIM and SH3 domain protein 1 OS=Homo sapiens GN=LASP1 PE=1 SV=2	1

	2.1	sp|P09651|ROA1	Heterogeneous nuclear ribonucleoprotein A1 OS=Homo sapiens GN=HNRNPA1 PE=1 SV=5	1
	2	tr|Q32Q12|Q32Q12	Nucleoside diphosphate kinase OS=Homo sapiens GN=NME1-NME2 PE=1 SV=1	1
	1.43	sp|Q07955|SRSF1	Serine/arginine-rich splicing factor 1 OS=Homo sapiens GN=SRSF1 PE=1 SV=2	1
F7	2	tr|K7EM90|K7EM90	Enolase (Fragment) OS=Homo sapiens GN=ENO1 PE=1 SV=1	1
	2	sp|P06733|ENOA	Alpha-enolase OS=Homo sapiens GN=ENO1 PE=1 SV=2	1
	1.6	sp|P84103|SRSF3	Serine/arginine-rich splicing factor 3 OS=Homo sapiens GN=SRSF3 PE=1 SV=1	1

F8	14.76	sp|P62937|PPIA	Peptidyl-prolyl cis-trans isomerase A OS=Homo sapiens GN=PPIA PE=1 SV=2	7
	12	tr|G3V1A4|G3V1A4	Cofilin 1 (Non-muscle), isoform CRA_a OS=Homo sapiens GN=CFL1 PE=1 SV=1	6
	12	tr|E9PK25|E9PK25	Cofilin-1 OS=Homo sapiens GN=CFL1 PE=1 SV=1	6
	11.46	sp|P04264|K2C1	Keratin, type II cytoskeletal 1 OS=Homo sapiens GN=KRT1 PE=1 SV=6	6
	6.11	sp|P13645|K1C10	Keratin, type I cytoskeletal 10 OS=Homo sapiens GN=KRT10 PE=1 SV=6	3
	5.82	sp|P17096|HMGA1	High mobility group protein HMG-I/HMG-Y OS=Homo sapiens GN=HMGA1 PE=1 SV=3	3
	5.04	tr|Q32Q12|Q32Q12	Nucleoside diphosphate kinase OS=Homo sapiens GN=NME1-NME2 PE=1 SV=1	4
	4.93	sp|P16949|STMN1	Stathmin OS=Homo sapiens GN=STMN1 PE=1 SV=3	2
	4.27	sp|Q93077|H2A1C	Histone H2A type 1-C OS=Homo sapiens GN=HIST1H2AC PE=1 SV=3	2
	4.27	sp|Q7L7L0|H2A3	Histone H2A type 3 OS=Homo sapiens GN=HIST3H2A PE=1 SV=3	2
	4.02	sp|Q8N257|H2B3B	Histone H2B type 3-B OS=Homo sapiens GN=HIST3H2BB PE=1 SV=3	2
	4.02	sp|P23527|H2B1O	Histone H2B type 1-O OS=Homo sapiens GN=HIST1H2BO PE=1 SV=3	2
	4	sp|P35908|K22E	Keratin, type II cytoskeletal 2 epidermal OS=Homo sapiens GN=KRT2 PE=1 SV=2	3
	2.84	sp|P62888|RL30	60S ribosomal protein L30 OS=Homo sapiens GN=RPL30 PE=1 SV=2	1
	2.61	sp|O60869|EDF1	Endothelial differentiation-related factor 1 OS=Homo sapiens GN=EDF1 PE=1 SV=1	2
	2.61	sp|O60869-3|EDF1	Isoform 3 of Endothelial differentiation-related factor 1 OS=Homo sapiens GN=EDF1	2
	2.61	sp|O60869-2|EDF1	Isoform 2 of Endothelial differentiation-related factor 1 OS=Homo sapiens GN=EDF1	2
	2.21	tr|B7Z6Z4|B7Z6Z4	Myosin light polypeptide 6 OS=Homo sapiens GN=MYL6 PE=2 SV=1	1
	2.08	sp|P07737|PROF1	Profilin-1 OS=Homo sapiens GN=PFN1 PE=1 SV=2	2
	2.08	tr|K7EJ44|K7EJ44	Profilin 1, isoform CRA_b OS=Homo sapiens GN=PFN1 PE=1 SV=1	2
	2.07	tr|J3QRS3|J3QRS3	Myosin regulatory light chain 12A OS=Homo sapiens GN=MYL12A PE=4 SV=1	1
	12.07	sp|O14950|ML12B	Myosin regulatory light chain 12B OS=Homo sapiens GN=MYL12B PE=1 SV=2	1
	2.02	sp|P60981|DEST	Destrin OS=Homo sapiens GN=DSTN PE=1 SV=3	1
	2.02	sp|P60981-2|DEST	Isoform 2 of Destrin OS=Homo sapiens GN=DSTN	1
	2	tr|E9PJK1|E9PJK1	Tetraspanin OS=Homo sapiens GN=CD81 PE=1 SV=1	1
	2	sp|P60033|CD81	CD81 antigen OS=Homo sapiens GN=CD81 PE=1 SV=1	1
	1.6	tr|U3KQK0|U3KQK0	Histone H2B OS=Homo sapiens GN=HIST1H2BN PE=1 SV=1	1
	1.48	sp|P17096-2|HMGA1	Isoform HMG-Y of High mobility group protein HMG-I/HMG-Y OS=Homo sapiens GN=HMGA1	2

1.3	tr|E9PP21|E9PP21_HUMAN	Cysteine and glycine-rich protein 1 OS=Homo sapiens GN=CSRP1 PE=1 SV=1	1
1.3	sp|P21291|CSRP1	Cysteine and glycine-rich protein 1 OS=Homo sapiens GN=CSRP1 PE=1 SV=3	1
1.21	sp|P30050|RL12	60S ribosomal protein L12 OS=Homo sapiens GN=RPL12 PE=1 SV=1	1
1.16	tr|H0YDD8|H0YDD8	60S acidic ribosomal protein P2 (Fragment) OS=Homo sapiens GN=RPLP2 PE=1 SV=1	1
1.16	sp|P05387|RLA2	60S acidic ribosomal protein P2 OS=Homo sapiens GN=RPLP2 PE=1 SV=1	1
0.84	sp|P24666-4|PPAC	Isoform 4 of Low molecular weight phosphotyrosine protein phosphatase OS=Homo sapiens GN=ACP1	1
0.84	tr|G5E9R5|G5E9R5	Acid phosphatase 1, soluble, isoform CRA_d OS=Homo sapiens GN=ACP1 PE=1 SV=1	1
0.81	sp|P24666|PPAC	Low molecular weight phosphotyrosine protein phosphatase OS=Homo sapiens GN=ACP1 PE=1 SV=3	1

Supplementary Method: SKOV-3 PEVs vs. EA.hy926 Challenge

The immortalized human endothelial cell line EA.hy926 was used, obtained from continuous culture in a 5% CO2 atmosphere, Dulbecco's Modified Eagle's Medium (DMEM) supplemented with 10% fetal bovine serum (FBS) and L-Glutamine. For the kinetics experiment, a total of $5x10^5$ EA.hy926 cells were seeded in 3-cm diameter Petri dishes in a volume sufficient to cover them with FBS-free DMEM medium. PEVs collected from SKOV-3 cells stimulated with the *E. coli* bacterial fraction were added to the cells at a 1:1 cell equivalence ratio (the total number of PEVs recovered from 1 SKOV-3 cell to 1 EA.hy926 cell). Images were taken at challenge kinetics of 0, 10, 20, 30, 40, 50, 60, and 90 min. During the interactions of EA.hy926 with SKOV-3 PEVs, some highly motile cells were observed, some cells secreting large EVs, and some cells detaching.

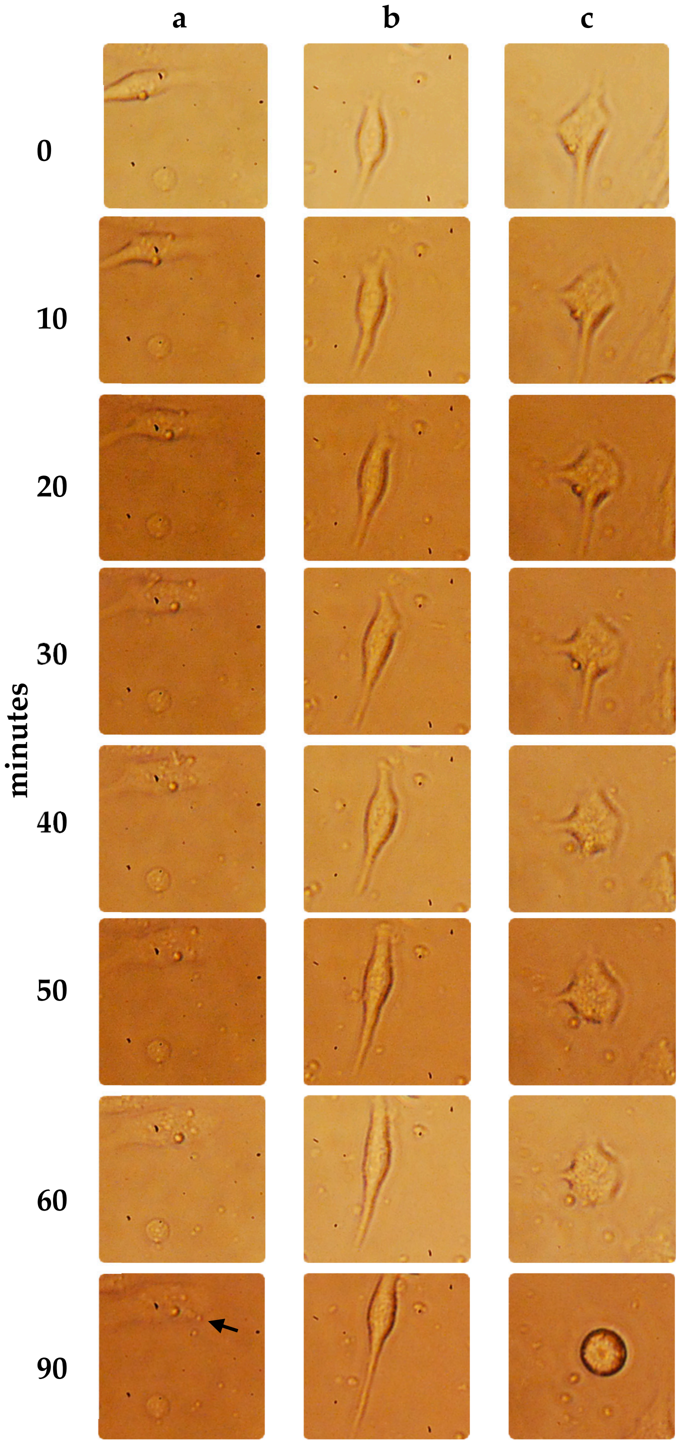

Figure S1. SKOV-3 PEVs vs. EAhy926 cells challenge. Cells were exposed to PEVs for 0 to 90 minutes. Three cells were selected to represent the observed behaviors. Some cells were observed secreting large EVs (a, arrow), others exhibiting dynamic motility or migration (b), and some detaching from the culture surface (c, 90 min).
